# Persistent accumulation of therapy-induced senescent cells: an obstacle to long-term cancer treatment efficacy

**DOI:** 10.1038/s41368-025-00380-w

**Published:** 2025-08-01

**Authors:** Jingjing Luo, Tongxu Sun, Zhenghui Liu, Yangfan Liu, Junjiang Liu, Shimeng Wang, Xueke Shi, Hongmei Zhou

**Affiliations:** 1 State Key Laboratory of Oral Diseases & National Center for Stomatology & National Clinical Research Center for Oral Diseases & Frontier Innovation Center for Dental Medicine Plus & West China Hospital of Stomatology, Sichuan University, Chengdu, China; 2 Department of Medicine, University of Colorado, Anschutz Medical Campus, Aurora, CO USA; 3 Department of Pathology and Laboratory Medicine, School of Medicine, University of California, Davis, Sacramento, CA USA

**Keywords:** Cancer therapy, Mechanisms of disease

## Abstract

In the ever-evolving landscape of cancer therapy, while cancer treatments such as chemotherapy, radiotherapy, and targeted therapy aim to eradicate malignant cells, they also inadvertently trigger cellular senescence in both cancerous and microenvironmental tissues. Therapy-induced senescence (TIS) can act as a barrier against tumor growth by halting cell proliferation in the short term, but the long-term persistence of therapy-induced senescent (TISnt) cells may pose a significant challenge in cancer management. Their distinct characteristics, like senescence-associated secretory phenotype (SASP), metabolic dysregulation, and immune evasion, make them exhibit remarkable heterogeneity to orchestrate the tumor microenvironment (TME), resulting in therapy resistance. However, how these TISnt cells functioning differently in cancer progression, and the intricate mechanisms by which they remodel the senescence-associated immunosuppressive microenvironment present challenges for improving anticancer therapy. Therefore, this review summarizes the heterogeneous TISnt cell phenotypes contributing to an accumulated senescent state, outlines their multidimensional interactions in the senescent microenvironment, and discusses current senescence-targeting strategies. Building on the current understanding of TIS, we propose potential avenues for improving TIS-targeting methodologies in the context of head and neck cancer, a representative heterogeneous malignancy, which can substantially enhance the efficacy of the “one-two punch” sequential treatment approach for head and neck cancer.

## Introduction

**Fig. 1:**
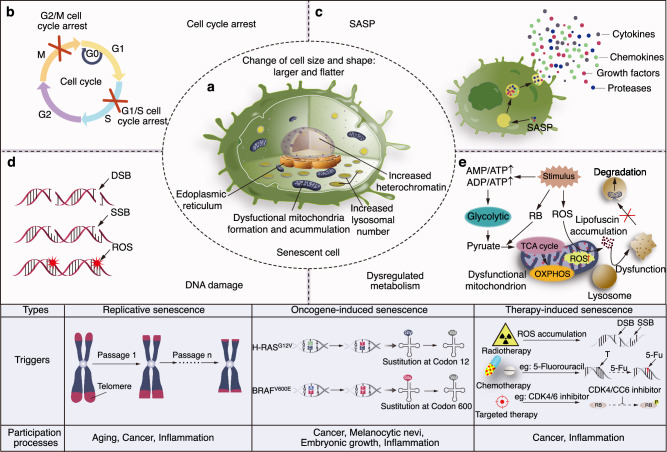
Hallmarks of senescent cells. **a** Senescent cell: A senescent cell is typically larger and flatter in shape, with nuclear heterochromatin and dysfunctional endoplasmic reticulum and mitochondria in cytoplasm. **b** Cell cycle arrest: As the classical phenotype of senescence, the cell cycle is generally arrested in G1 phase, and a few cells are arrested in G2 phase. **c** SASP: The senescence-associated secretory phenotype (SASP) represents hypersecretion of multiple cytokines, growth factors, chemokines and proteases by senescent cells. **d** DNA damage: The most common trigger of cellular senescence is DNA damage, including DNA double-strand breaks (DSBs), DNA single-strand breaks (SSBs) and oxidative injury; in turn, it is also the outcome index used to detect senescence. **e** Dysregulated metabolism: Upon exposure to senescence-related stimuli, the ratios of AMP/ATP and ADP/ATP in senescent cells increase, promoting glycolysis activity to provide ATP for active secretion. Dysfunctional mitochondria further increase reactive oxygen species (ROS) levels, leading to lipofuscin formation to accumulate lysosomes. These lysosomes struggle to clear dysfunctional mitochondria, leading to further damage. Lowest panels: Together with therapy-induced senescence (TIS) caused by DNA damage from radiotherapy, chemotherapy and targeted therapy in cancers, the other common types of senescence include replicative senescence (RS) triggered by telomere shortening during continuous cell proliferation, and oncogene-induced senescence (OIS), as represented by H-RASG12V and BRAFV600E mutations in codon 12 and codon 600. *Created with Adobe Illustrator Artwork*

Therapy-induced senescence (TIS) is a form of cellular senescence triggered by cancer therapeutics, such as radiotherapy, chemotherapy or targeted therapy. This process arises from nonlethal DNA damage, leading to cell cycle arrest—a hallmark shared with replicative senescence and oncogene-induced senescence (Fig. 1). In the short term, therapy-induced senescent (TISnt) cells act as a barrier against carcinogenesis and a booster facilitating anticancer treatments in the short term. In some cases, prosenescent antitumor therapeutics^1^ are strategically crafted to impede cancer progression by activating specific prosenescent effectors,^2^ and inhibiting telomerase to amplify the senescent response.^3^ However, a growing body of evidence indicates that accumulated TISnt cells within tumor microenvironment (TME) may exert paradoxical effects, promoting therapy resistance, tumor relapse and metastasis.

Research indicates that therapy-resistant cancer cells can evade treatment by exploiting the DNA damage repair response, either by entering senescence or easily escaping proliferation arrest to adopt more malignant behavior upon recovery.^4,5^ TIS of cancer cells also constitutes a critical mechanism of treatment resistance in head and neck squamous cell carcinoma (HNSCC). Senescent HNSCC cells that escape proliferative arrest exhibit enhanced heterogeneity and correlate with poor prognosis.^6,7^ Cisplatin-based chemotherapy has been experimentally confirmed to induce cellular senescence in HNSCC. Therapeutically, senolytic agents may be employed to eliminate residual senescent tumor cells post-chemotherapy, potentially delaying disease recurrence in HNSCC patients.^8,9^ Meanwhile, benign stromal and immune cells within the TME—such as cancer-associated fibroblasts (CAFs) and tumor-associated macrophages (TAMs)—often display a sublethal or senescent status due to their limited proliferation capacity,^10^ leading to a certain insensitivity to cancer treatment.^11^ These diverse senescent cells possess the capacity to sustain and intensify the TISnt state through well-established mechanisms such as senescence-associated secretory phenotype (SASP), mitochondrial dysfunction or altered cellular communications,^12^ along with unique pathways involving specific cell types.^13^ In the context of HNSCC, radiation therapy induces a distinct SASP that drives critical therapy resistance through NFκB-dependent signaling,^14,15^ while radiation-induced senescent CAFs maintain the ability to promote radioresistance in oral squamous cell carcinoma (OSCC) cells even when their own proliferation is inhibited.^16^ Accordingly, these TISnt cells—both malignant and microenvironmental—engage in dynamic interactions that lead to progressive accumulation and microenvironmental remodeling, ultimately establishing a senescent microenvironment.^17^ This persistent senescent state, whether during sequence therapy or for an extended period post-treatment, has been proposed as a potential mechanism for sustaining minimal residual disease, which may foster tumor recurrence or metastasis.

The senescent TME often manifests chronic inflammatory and immunosuppressive characteristics.^18–20^ This process is primarily mediated by SASP, which is highly heterogeneous and dynamic dependent on the activation of specific cell types.^21–23^ The effects of the SASP extend beyond the senescent cells themselves, influencing neighboring immune and mesenchymal cells.^24–26^ The propagation of cellular senescence, akin to an “infectious” process, significantly contributes to the establishment of persistent senescence within the tumor microenvironment.^27,28^ Further, their interactions lead to the recruitment of immunosuppressive cell populations, such as regulatory T cells (Tregs) and myeloid-derived suppressor cells (MDSCs), thereby facilitating immune evasion by cancer cells and promoting tumor progression. The accumulation of TISnt cells prolongs chronic inflammation and immunosuppression within the TME.^18,29,30^ HNSCC employs unique immune evasion mechanisms that confer resistance to conventional and even combination therapies. These mechanisms range from direct T-cell inhibition via soluble or surface-expressed inhibitory factors to recruitment of immunosuppressive cell populations.^31^ For instance, immunosuppressive cytokines including transforming growth factor (TGF)-β, interleukin (IL)-6, and IL-10 potently inhibit T-cell proliferation and effector functions. Notably, these same cytokines contribute to the establishment and maintenance of the senescent tumor microenvironment while simultaneously antagonizing therapeutic efficacy in HNSCC.^32^

Therefore, the recognition of the heterogeneous and detrimental nature of TISnt cells within the TME, has indeed become a significant focus in cancer research and therapeutics. This review summarizes the diverse phenotypes of TISnt cells that accumulate within the TME, examines the intricate mechanisms through which they interact with each other to sustain a persistent senescent state, and reviews current and emerging approaches targeting TISnt cells to effectively counteract their adverse effects on cancer treatment. Particularly, we anticipate providing more information for developing a “one-two punch” sequential strategy that combines conventional anticancer therapeutics with TIS-targeting methods particularly for head and neck cancer (HNC), thereby improving overall treatment outcomes.

## Heterogeneity Of therapy-induced senescent cells

Notably, anticancer therapy can trigger senescence in various cell types, including both cancerous and non-cancerous cells. Within the dynamic TME, diverse cell populations and signaling molecules intricately interact.^33,34^ This inherent complexity of the TME contributes to the distinctive heterogeneity observed in both TISnt cells and their molecular characteristics. Furthermore, heterogeneity is modulated by specific types of anticancer therapies. Henceforth, we will delve into the critical cellular constituents and senescence-associated molecular profiles within tumors, contextualized within prevalent DNA damage-inducing anticancer treatments, including radiotherapy, chemotherapy, and targeted therapy (specific features are also summarized in Table 1).

**Table 1:** Diverse therapy induces senescent cells in tumors and the surrounding microenvironment

Cell type	Origin	Therapy	Main TIS Characteristics	Refs
Cancer cells	Head and neck cancer	Cisplatin	SA-β -Gal ↑ , P53 ↑ , p21^WAF1^ ↑ , IL-6 ↑ , IL-8 ↑ , IL-1β ↑	^8^
		Palbociclib	SA-β -Gal ↑ , morphological changes	^9^
		IR	SA-β -Gal ↑ , SASP factors (CXCL1, CXCL3, CXCL8, CXCL10, CXCL11, IL-1α, IL-1β, MIF, VEGF) ↑	^15^
	Breast cancer	IR	SA-β -Gal↑	^39^
		Doxorubicin	SA-β -Gal ↑ , recovered metabolism	^51^
	Non-small cell lung cancer	IR	BrdU (-), SA-β -Gal ↑ , p16^INK4a^ ↑ , p53/p21 pathway activated	^41^
	Colon, cervical, lung adenocarcinoma, and fibrosarcoma	IR	SA-β -Gal ↑ , multiple nuclei and micronuclei	^44^
	Lymphoma	cyclophosphamide	P53 ↑ , p16^INK4a^ ↑	^47^
	Lung cancer/breast cancer	Neoadjuvant chemotherapy	SA-β -Gal↑	^48,49^
	Lymphoma	Cyclophosphamide/doxorubicin	BrdU (-), Ki67 ↓ , SA-β -Gal ↑ , glucose utilization ↑ , ATP production↑	^50^
	Melanoma	Cisplatin	SA-β -Gal ↑ , γ-H2AX foci ↑ , P53 ↑ , p21^WAF1^ ↑ , SASP factors (IL-1α、IL-1β、IL-6、IL-8 and TNF-α)↑	^46^
	Lung and pancreatic cancer	CDK4/6 inhibitor +MEK inhibitor	SASP factors (CCL2, CCL4, CCL5, CXCL10, CX3CL1, IL-15, IL-18, TNF-α) ↑	^53^
CSC	Malignant pleural mesothelioma	Pemetrexed	IL-6 ↑ , IL-8 ↑ , VEGF-A ↑ , IFNγ ↑ , CXCL1, PlGF1 mRNAs↑	^65,66^
	Liver cancer	Doxorubicin	IL-6 ↑ , TGF-β ↑ , p16 ↑ , p21 ↑ , P53↑	^66^
Fibroblasts	Fibroblasts from normal human lung	IR	SA-β -Gal ↑ , p21^WAF1^ ↑ , p16^INK4a^↑	^75^
	Primary cultured fibroblasts from human skin	IR	SA-β -Gal ↑ , p16^INK4a^ ↑ , γ-H2AX foci ↑ , atypical MHC molecules (MICA/B and HLA-E) ↑,	^89^
	Fibroblasts from normal mouse/ human	Doxorubicin	SA-β -Gal ↑ , p16^INK4a^ ↑ , SASP factors (IL-1α, IL-6, MMP-3, MMP-9, CXCL1, CXCL10 and CCL20) ↑	^76^
	Fibroblasts from normal human	Bleomycin	SA-β -Gal ↑ , AREG ↑ , SASP factors (HGF, IL-1β, IL-1α, MCP-1, MMP-1, MMP-2, MMP-3) ↑	^71,77^
	Primary cultured CAFs from human lung	IR	SA-β -Gal ↑ , 53BP1 foci ↑ , reduced migration and invasion	^84^
	Primary cultured CAFs from human colorectal cancer	IR	P53 ↑ , p21^WAF1^ ↑ , insulin-like growth factor-1↑	^85^
	Primary cultured CAFs from HNSCC	IR	SA-β -Gal ↑ , BrdU (-), 53BP1 foci↑	^16^
Endothelial cells	Endothelial cells from human umbilical vein	IR/doxorubicin	SA-β-Gal ↑ , pRb phosphorylation ↓ , P53/P21 ↑ , SASP factors (CXCL11) ↑	^92^
		Sunitinib	SA-β -Gal ↑ , p16^INK4a^ ↑ , P53 ↑ , P57 ↑ , SASP factors (CCL6, complement component C5a, chemerin, IL16) ↑	^93^
Immune cells	NK cells from healthy individuals	IR	CD57(-), NKG2A ↑ , morphological changes	^95–97^
	Neutrophils from breast cancer	Chemotherapy	Elevated exosomes, oversegmented nuclei, disrupted metabolism, potent immunosuppressive capabilities, SIRT1↑	^99,100^
	Macrophages from C57BL/6 J mice	IR	SA-β -Gal ↑ , p16^INK4a^ ↑ , p21^WAF1^ ↑ , BCL-2 ↑ , BCL-XL ↑ , SASP factors (TGF-β1, Arg-1, IL-1α, IL -6, TNF-α, CCL2, CXCL10, CCL17, MMP2, MMP9, MMP12) ↑	^103^
	CD8 + T cells from C57BL/ 6 mice	IR	Proliferation ↓ , metabolism↓	^104^
	CD3 + T cells from hematopoietic cell transplantation	Chemotherapy	Number in peripheral blood ↓ , p16^INK4a^ ↑	^105^

### Cancer cells: senescence is susceptible to diverse therapies

The therapeutic response of cancer cells directly determines the short-term anticancer effect of various treatments. Ideally, increased DNA damage resulting from cancer therapy leads to cell death, whereas nonlethal DNA damage preferentially induces cellular senescence. This is particularly relevant in the context of chemotherapy or radiotherapy, where single doses may be modulated to mitigate toxic side effects, inadvertently promoting cellular senescence over cell death.^35,36^

Radiotherapy, which utilizes ionizing radiation (IR) to cause DNA double-strand breaks (DSBs), is a potent way to stimulate cellular senescence^37^; therefore, IR-induced senescent cancer cells have been widely studied.^38–41^ The radiosensitivity of cancer cells determines whether they become senescent or dead after exposure to a specific IR dose. It has been reported that when the IR dose is less than 10 Gy, cancer cells tend to undergo cellular senescence, whereas higher doses typically induce cell apoptosis.^42,43^ Research involving various cancer cell lines, has revealed that most DNA double-strand breaks (DSBs) induced by IR doses below 10 Gy can be repaired, leading to the survival of senescent cancer cells. Moreover, an increase in senescence-associated β-galactosidase (SA-β-Gal) expression was observed before cell death,^44^ suggesting that IR-induced senescence may be widespread and dynamic in cancer cells. IR can also induce the expression of p16^INK4a^ and maintain DNA damage in both human and mouse models for several months,^45^ indicating the long-term existence of TIS under real-world conditions.

Various genotoxic and cytotoxic drugs commonly used in chemotherapy, such as cyclophosphamide, carboplatin, Taxol, and cisplatin, have also been reported to induce cellular senescence in cancer cells. Chemotherapy-induced senescence, as evidenced by the significant upregulation of P53, SA-β-Gal, and SASP factors, has been observed in melanoma cells,^46^ in a lymphoma mouse model,^47^ and in human biopsies of breast and lung cancer patients.^48,49^ Another report indicated that chemotherapy-induced senescent lymphoma cells obtained increased glucose utilization and ATP production, revealing a greater metabolic characteristic of TISnt cells.^50^ Similarly, although a decrease in the overall metabolism of senescent breast cancer cells occurred shortly after doxorubicin treatment, metabolic pathways related to energy, amino acid, and lipid metabolism recovered synchronously as cells transition to a stable senescence state.^51^

Molecular drugs targeting the cell cycle of cancer cells may directly promote cellular senescence, representing a specific type of TIS. For instance, cyclin-dependent kinase 4 and 6 (CDK4/6) inhibitors have been demonstrated to induce dormancy or senescence in various cancer cells.^52^ Combination therapies, such as MEK inhibitors with CDK4/6 inhibitors, have also been shown to induce durable proliferation arrest and to upregulate SASP factors, including tumor necrosis factor-α (TNF-α) and intercellular adhesion molecule-1 (ICAM-1).^53^ Additionally, inhibitors of Aurora kinase, which disrupts mitotic spindle assembly and mitotic progression, have been found to block cancer cell proliferation and induce cellular senescence in glioblastoma, melanoma, and pancreatic cancer.^54–56^ Inhibition of the DNA replication kinase CDC7 has also been shown to selectively induce senescence in TP53-mutant liver cancer cells.^57^ Furthermore, poly (ADP‒ribose) polymerase (PARP) inhibitors, known as synthetic lethal anticancer drugs, can induce senescence in ovarian and breast cancer cells, characterized by increased SA-β-gal and SASP factors of IL-6 and IL-8.^58^

### Cancer stem cells: unclear transition to senescence in response to cancer treatment

Cancer stem cells (CSCs), with their robust self-renewal capabilities, act as the seeds of cancer and significantly impacts treatment outcomes.^59–61^ These CSCs, which are typically dormant, exhibit inherent resistance to various anticancer therapies. Until recently, some researchers considered that CSCs were unlikely to undergo senescence, even in the presence of accumulated genetic damage.^62^ However, emerging evidence challenges this notion, revealing that CSCs can indeed enter a senescent state characterized by increased cell volume, positive SA-β-gal, and elevated expression of SASP.^63^ Bone morphogenetic protein 7 (BMP7), which is secreted by stromal cells, has been shown to trigger the senescence of prostate CSCs through SA-β-Gal activity and increased expression of p21.^64^ In addition, purified ALDH^high^ mesothelioma cells, a subpopulation of CSCs, maintain a SASP-induced phenotype through both cell-autonomous and cell-nonautonomous mechanisms.^65^ A subpopulation of breast CSCs becomes activated and enters the cell cycle following exposure to 3 Gy irradiation for five consecutive days, accompanied by increased SA-β-Gal activity.^39^ A subset of liver CSCs characterized by epithelial cell adhesion molecule (EpCAM)/CD133 positivity displayed a senescent phenotype in response to doxorubicin treatment. These senescent CSCs exhibited increased SASP secretion of IL-6 and TGF-β, along with upregulation of senescence-related genes such as p16, p21, and p53. Interestingly, despite showing features of senescence, these cells still maintained the expression of stem-reprogramming genes such as SOX2, KLF4, and c-MYC.^66^

Further, some evidences suggest that polyploid giant cancer cells (PGCCs) may represent a unique type of senescent CSC characterized by senescence, such as a flattened shape and SA-β-Gal expression, and stemness, such as tumor-initiating capacity.^67,68^ Adult stem cells, which serve as potential precursors of CSCs, may be driven into senescence by IR, resulting in impaired differentiation function and altered biological behavior rather than permanent proliferation arrest and expression of common markers of cellular senescence. These findings provide indirect evidence suggesting that the senescent phenotype of CSCs may involve atypical cell cycle arrest and dysfunctional differentiation. Overall, it remains uncertain whether senescent CSCs arise directly in response to cancer therapy. However, when CSCs activated by treatment and re-entered the cell cycle,^39,69^ the continuous treatment could induce these activated CSCs to proliferate and acquire a senescent phenotype similar to that of differentiated cancer cells.

### Fibroblasts: resistant to cancer therapy and sensitive to cellular senescence

Fibroblasts are the most common stromal cell type within the TME. Cellular senescence was first observed in lung fibroblasts by Hayflick and Moorhead.^70^ Studies have consistently demonstrated that these senescent fibroblasts exert a significant tumor-promoting effect.^71–73^ Following exposure to IR, human normal fibroblast lines have been observed to undergo long-term stagnation of proliferation.^74^ Subsequent studies have further demonstrated that lung fibroblasts can survive 50 Gy irradiation, displaying a pronounced senescent phenotype characterized by increased cell volume, elevated SA-β-Gal activity, and substantially upregulated expression levels of p21^WAF1^ and p16^INK4a^.^75^ Similarly, mouse and human dermal fibroblasts are induced to undergo senescence in response to chemotherapeutic drugs, such as doxorubicin, paclitaxel, temozolomide, and cisplatin. These senescent fibroblasts exhibit substantially decreased DNA synthesis, increased expression of p16^INK4a^ mRNA, and elevated levels of numerous SASP components, including IL-1α, IL-6, matrix metalloproteinase-3 (MMP-3), MMP-9, C-X-C motif chemokine 1 (CXCL1), CXCL10, and chemokine (C-C motif) ligand 20 (CCL20).^76^ Furthermore, bleomycin-induced senescent fibroblasts exhibit increased paracrine secretion of various growth factors and MMPs.^71,77^

Some studies have shown that the SASP of senescent fibroblasts, including IL-6, IL-8, and a variety of CXCLs, is similar to that of the paracrine components secreted by CAFs. This finding suggests that senescent fibroblasts may represent a functional subtype of CAFs.^78^ CAFs are known to promote tumor progression through their paracrine actions and interactions with cancer cells.^79–83^ Several studies have examined the response of senescent CAFs to anticancer therapy. For instance, in non-small cell lung cancer (NSCLC), IR-induced senescent CAFs exhibit increased SA-β-gal activity along with irreparable DNA damage detected by 53BP1 foci.^84^ Similarly, in colorectal cancer, IR-induced senescent CAFs displayed p53 activation and increased the secretion of insulin-like growth factor-1 (IGF1), which contributed to cancer progression.^85^ Our previous study focusing on oral cancer revealed that a single dose of 18 Gy induced senescence in CAFs characterized by proliferation blockade, permanent DNA damage, and increased SA-β-Gal activity.^16^ The long-term viability of these senescent CAFs poses a risk of reducing radiosensitivity of oral cancer cells, leading to their characterization as “zombie-like” CAFs due to their detrimental effects. This notion is supported by other researchers, who have referred to the persistent senescent cells as “zombies” because of their accumulation resulting in disease progression.^86,87^

Additionally, fibroblasts play a crucial role in tumor progression by interacting with various immune cells within the TME. Recent studies have further proposed the role of fibroblasts as sentinel cells in certain immune responses.^88^ IR-induced senescent human fibroblasts express atypical major histocompatibility complex (MHC) molecules. This expression pattern aids senescent cancer cells in evading immunological detection by natural killer (NK) cells and CD8 + T cells.^89^ These findings highlight the significant immunomodulatory effects of senescent fibroblasts, underscoring their crucial role in maintaining the immunosuppressive microenvironment associated with TIS.

### Endothelial cells: easily undergo senescence with paradoxical effects on anticancer therapy

Endothelial cells (ECs) form a delicate epithelial layer lining the inner walls of blood vessels.^90^ Within TME, tumor-associated ECs play crucial roles in angiogenesis, maintaining barrier function, and mediating paracrine signaling within tumors.^91^ Theoretically, because they are epithelial in nature, ECs are susceptible to entering a senescent state induced by cancer therapy. For instance, exposure to 6 Gy of IR leads to senescence in various types of endothelial cells, characterized by low phosphorylation of pRb, accumulation of p53/P21, increased SA-β-Gal activity, and elevated secretion of multiple inflammatory chemokines.^92^ In this study, low concentrations of doxorubicin (e.g., 50 ng/mL) were also reported to induce senescence in the majority of ECs.

Given that ECs are responsible for forming vasculature within tumors, senescent ECs might inhibit cancer progression by impairing tumor angiogenesis. This is because senescent endothelial cells are less likely to support the formation of new blood vessels, which are crucial for tumor growth and metastasis. However, senescent ECs induced by the antiangiogenic drug sunitinib, have been found to have their potential pro-cancer effects.^93^ These TISnt endothelial cells secrete increased levels of SASP components, including CCL6, complement C5a and IL-16. This creates an inflammatory microenvironment that promotes the migration and invasion of breast cancer cells, thereby increasing the risk of distal metastasis. Even in the absence of the typical SASP, senescent ECs induced by the chemotherapeutic agent doxorubicin release acute levels of IL-6 through reactive oxygen species (ROS)-mediated p38 activation, promoting chemoresistance.^94^ Additionally, senescent ECs have been shown to induce the formation of dysfunctional vessels with increased permeability within the TME. These dysfunctional vessels may facilitate the intravasation and extravasation of metastatic cancer cells, contributing to the spread of cancer.^93^

### Immune cells: nonclassical cells with a specific senescent phenotype

In the context of anticancer therapy, immune cells can also exhibit a senescent phenotype leading to functional impairment and shortcomings in cancer treatments. NK cells, as one of the important innate immune cells, monitor and kill cancer cells. A specific senescence phenotype of NK cells, was characterized by a lack of CD57 expression, a maturation marker commonly used to identify late-differentiated senescent cells,^95^ and an increase in the expression of NKG2A, an inhibitory receptor that typically decreases the number of mature NK cells. These senescent NK cells do not exhibit differences in either cell proliferation or interferon-γ (IFN-γ) production or cell cytotoxicity.^96^ However, persistent CD158d activation, which inhibits an NK-activated receptor, can induce NK cells to exhibit all hallmarks of senescence by causing DNA damage, including morphological changes, survival without entering the cell cycle, and significant secretion of SASP components.^97^ Additionally, NK cells exhibit irradiation tolerance up to 30 Gy, with potential partial dysfunction with increasing IR, although they still retain some cytotoxic activity.^10,98^

Some scholars have elucidated the occurrence of senescent neutrophils in breast cancer patients undergoing chemotherapy.^99^ These senescent neutrophils specifically exhibit elevated exosome production in a STAT3-dependent manner, consequently upregulating the expression of adiposity- and obesity-associated proteins (FTOs) in breast cancer cells via piRNA-17560. Moreover, a scarcely explored subset of tumor-associated aged neutrophils (Naged), has been identified as those with CXCR4^+^CD62L^low^ expression, and as a significant contributor to breast cancer lung metastasis.^100^ These cells are completely different from non-aged or inflammation-associated aged neutrophils, as they exhibit over-segmented nuclei, disrupted metabolism, potent immunosuppressive capabilities, and heightened SIRT1 expression, which aids in prolonged survival.

Mature macrophages and dendritic cells (DCs) stimulated with granulocyte-macrophage colony-stimulating factors (GM-CSF) and IL-4, demonstrate a robust capacity to repair DSBs induced by anticancer agents, such as temozolomide, irradiation and oxidative agents. Their precursor cells, monocytes, are sensitive to DNA damage accumulation and deficiencies in DNA repair, often leading to apoptosis.^101,102^ This observation suggested that these DNA damage-resistant macrophages and DCs may be prone to entering a state of TISnt status rather than undergoing cell death. Indirect evidence supporting this notion comes from a radiation-induced pulmonary fibrosis model in which IR-induced senescent macrophages displayed elevated expression levels of senescence markers such as SA-β-Gal and senescence-related genes, including P16, P21, BCL-2, and BCL-XL. These senescent macrophages exhibit significantly increased secretion of proinflammatory factors, chemokines, and matrix metalloproteinases—characteristic features of the SASP.^103^

Compared with the innate immune response, the adaptive immune response in cancer is highly specific for targeting cancer cells. Lymphocytes, including CD8 + T cells, are particularly sensitive to the toxic effects of cancer therapy compared to other cell types within the TME. Even low doses of IR, such as 3 Gy, have been shown to significantly decrease the proliferation of CD8 + T cells and reduce cytokine production upon T cell receptor (TCR) stimulation.^104^ Additionally, significant upregulation of p16^INK4^ expression was observed in CD3 + T cells following chemotherapy and stem cell transplantation in some patients. These senescent T cells exhibit a bias toward the expression of transcripts associated with CD8 + T and NK cells.^105^ This phenomenon has also been observed in breast cancer patients after chemotherapy, where senescent CD8 + T cells were detected in the tumor microenvironment.^106^

## The dark role and molecular mechanism of therapy-induced senescence in compromising cancer therapy

**Fig. 2:**
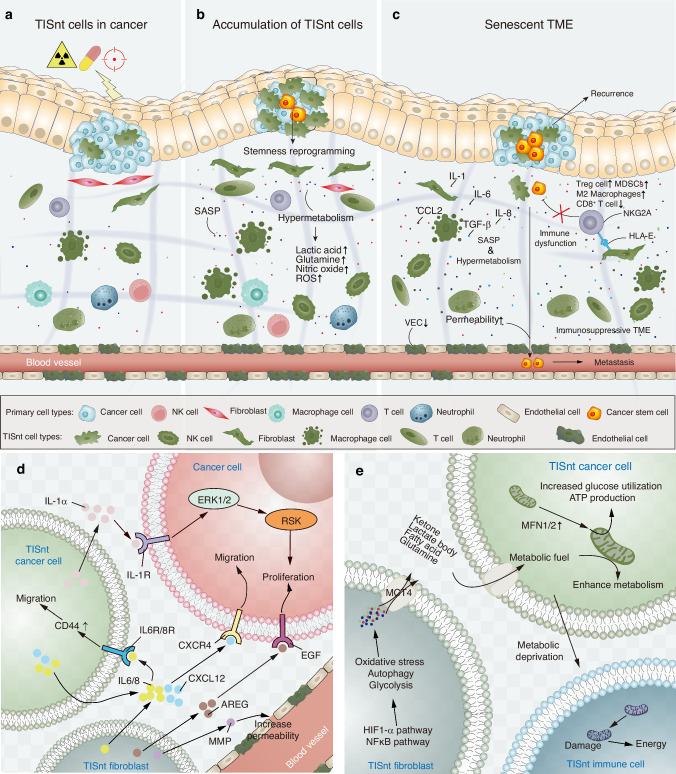
The emergence, accumulation and interaction of TISnt cells contributing to a senescent microenvironment. **a** Anticancer therapies, through the induction of DNA damage, have been shown to elicit various forms of cellular senescence within both the tumor and its surrounding microenvironment. These TISnt cells exhibit a complex and heterogeneous phenotype. **b** Over the course of treatment, TISnt cells, along with their associated SASP and metabolites, gradually accumulate. A subset of TISnt cancer cells evade senescence and undergo reprogramming, acquiring stemness characteristics. **c** As TISnt cells accumulate, a senescent TME manifests characterized notably by immunosuppressive features. Under such conditions, the presence of resilient cancer cells or reprogrammed CSCs poses a heightened risk of tumor recurrence and metastasis. Additionally, senescence-associated increases in vascular permeability may further facilitate distant metastasis. **d** Complicated SASP-dependent crosstalk between TISnt cells and non-senescent cancer cells directly promotes the proliferation and migration of cancer cells or indirectly promotes tumor metastasis by increasing vascular endothelial permeability. **e** The high metabolism of TISnt cancer cells is further promoted by mitochondrial energy output from TISnt fibroblasts and facilitates the dysfunction of TISnt immune cells with mitochondrial damage through metabolic deprivation. *Created with Adobe Illustrator Artwork*

The phenomenon of TISnt cells is intricate and multifaceted. The dark side of these cells has gradually come to light. Elucidating the underlying mechanisms governing the emergence of TISnt cells and their persistent accumulation has become imperative for the advancement of novel therapeutic strategies aimed at combating cancer. Herein, we propose a comprehensive summary of five putative mechanisms underlying TISnt cell-mediated impediments to cancer therapy efficacy: SASP, metabolic dysregulation, immune dysfunction and evasion, senescence escape and stemness reprogramming, and an accumulated senescent state. These mechanisms collectively present formidable challenges that must be addressed to enhance the efficacy of cancer therapies (Fig. 2).

### The deleterious effect of SASP

The SASP characterizes senescent cells, showcasing a secretome encompassing numerous bioactive molecules, notably proinflammatory cytokines, growth factors, proteases, and protease inhibitors.^21,107,108^ It is believed to foster a persistent, chronic, and inflammatory microenvironment, perpetuate chronic inflammation, drive angiogenesis, and enhance cancer cell aggressiveness, thereby fueling cancer progression and metastasis.^22,26^ As typical components of the SASP and key mediators of the inflammatory response, IL-1 and IL-6 are pivotal in triggering and promoting inflammatory reactions to aggravate cancer progression.^109^ IL-32 and IL-11, also identified as proinflammatory factors in the SASP, contribute to maintaining the inflammatory environment, thereby facilitating cancer progression.^110,111^ Exogenous administration of the cytokines IL-6 and IL-8 in vitro induced a senescent phenotype in MCF-7 cells, a low invasive breast cancer cell line, thereby enhancing their migration and self-renewal ability. This induction subsequently leads to the upregulation of IL-6 and IL-8 secretion, establishing an autocrine loop.^112^ Consistently, in cisplatin-induced senescent melanoma cells, the ERK1/2-RSK1 pathway is activated through IL-1α expression to promote the proliferation of nonsenescent cells and increase IL-8 secretion,^46^ thus synergistically amplifying the SASP effect. Senescent papillary thyroid carcinoma (PTC) cells exhibit enhanced invasion potential attributed to elevated expression of IL-6, IL-8, and MMPs. These cells facilitate the recruitment of non-senescent cancer cells by activating the CXCL12/CXCR4 signaling pathway, consequently promoting collective invasion in PTC.^113^ Apart from the intrinsic secretion of TISnt cancer cells, SASP factors originating from the surrounding TME predominantly exert deleterious effects on cancer cells, with a significant portion derived from senescent fibroblasts. For instance, senescent prostate fibroblasts induced by bleomycin exhibit elevated levels of fibroblast growth factor 7 (FGF7), hepatocyte growth factor (HGF), and amphiregulin (AREG), all of which increase the proliferation of cancer cells.^71^ Senescent mouse fibroblasts induced by adriamycin/paclitaxel treatment aggravated the side effects of cancer treatment, including inflammation, cardiac dysfunction, severe fatigue, and tumor recurrence and metastasis. These effects are mediated by secreted SASP proteins, such as IL-1α, IL-6, and CXCL1.^76^ IR-induced senescent skin fibroblasts were also shown to increase sprouting angiogenesis through SASP induced by IL-6 overexpression in 3D microvessel-on-a-chip-containing fibroblasts.^114^ Senescent fibroblasts in human breast cancer showed upregulation of MMPs, accompanied by increased permeability of adjacent capillaries, exposing cancer cells to higher levels of plasma products.^77^ Furthermore, treatment with a CDK4/6 inhibitor in melanoma resulted in increased SASP-associated transcripts, such as IL-6, MMP3, CCL6, CCL8, and CCL11, in senescent fibroblasts. This upregulation enhanced melanoma cell proliferation and tumor growth in vivo, along with activation of NF-κB signaling.^115^

The amount of SASP compounds released from TISnt endothelial cells, such as the upregulation of CXCL11 and the secretion of a large amount of proinflammatory cytokines, has also been reported to promote the aggressiveness of breast cancer cells.^92^ Sunitinib has been shown to increase the expression of SASP components in senescent endothelial cells. These components included inflammatory chemokines, such as CCL6, complement component C5a, chemerin, and IL-16. Their increase in SASP production contributes to the enhanced recruitment of breast cancer cells to vessels, thereby facilitating cancer cell migration.^93^ The understanding of the TIS-related SASP originating from immune cells within the TME remains limited. One study indicated that CD158d-stimulated senescent NK cells secreted SASP factors, promoting vascular remodeling and angiogenesis at sites expressing HLA-G.^97^ IR-induced senescent macrophages in pulmonary fibrosis exhibit increased SASP expression of profibrogenic and proinflammatory factors, chemokines, and matrix metalloproteinases.^103^ These findings suggest the potential SASP of TISnt immune cells can consequently impact treatment efficacy.

### Metabolic dysregulation

Senescent cancer cells often exhibit a characteristic hypermetabolic response, manifested as heightened metabolic activity, notably increased glucose consumption and lactate production.^50,116^ In a murine lymphoma model, tumor cells displaying TIS demonstrated augmented glucose uptake, enhanced fatty acid catabolism, and elevated ATP generation through oxidative phosphorylation. This metabolic shift was attributed to proteotoxic stress and the accumulation of SASP components following cyclophosphamide and adriamycin chemotherapy. These effects collectively activated an endoplasmic reticulum (ER) stress response, initiating the unfolded protein response (UPR) and triggering a ubiquitination-mediated autophagic cascade in senescent cells.^50^ Investigations in melanoma have elucidated the reciprocal relationship between metabolic alterations and SASP. TISnt melanoma cells exhibit increased mitochondrial mass and length, accompanied by increased energy metabolism and coupling efficiency.^117^ This increase in mitochondrial energy metabolism mediated by the mitochondrial fusion proteins mitofusin 1 and 2 (MFN1 and 2) significantly potentiated the secretion of IL-6, a key element of the SASP. Furthermore, senescence-associated HMGAs regulated NAD+ metabolism through enhanced glycolysis and mitochondrial energy metabolism, thereby governing the proinflammatory SASP.^118^ Additionally, it has been reported that human proliferative cells experiencing mitochondrial dysfunction-associated senescence exhibit reduced NAD + /NADH ratios, thereby leading to a modified SASP that lacks the IL-1-dependent inflammatory arm. This alteration occurs through AMP-activated protein kinase (AMPK)-mediated activation of p53.^119^ In breast cancer cells treated with estrogen, mitochondrial dysfunction can induce a senescent phenotype characterized by mitochondrial membrane depolarization and increased accumulation of flattened lysosomes and mitochondria. This phenomenon is accompanied by elevated expression levels of LC3-I/II, PTEN-induced putative kinase 1 (PINK1), and lysosome-associated membrane protein 2 (LAMP2).^120^ Furthermore, ER stress represents another metabolic trigger of TIS.^121^ SASP-producing TISnt lymphoma cells demonstrate ER stress, the UPR, and increased ubiquitination. These TISnt lymphomas are susceptible to interventions blocking glucose utilization, leading to selective elimination through ER-related apoptosis mediated by caspase-12 and caspase-3.^50^ Notably, augmented glutamine intake by cancer cells provides a nitrogen source for nucleotide synthesis, aiding in evasion of the TIS state and acquisition of CSC-like properties.^122^

Stromal cells within the TME play a pivotal role in providing nutrients and energy to cancer cells. Senescent stromal cells exhibit a unique metabolic reprogramming phenotype characterized by pyruvate dehydrogenase kinase 4 (PDK4) -dependent upregulation of aerobic glycolysis and elevated lactate secretion, while preserving mitochondrial respiration and redox homeostasis. Notably, the accumulated lactate stimulates ROS generation through NOX1 activation, thereby driving the SASP.^123^ Notably, senescent CAFs present in various cancers, contribute to the establishment of a nutrient-rich microenvironment that supports tumor growth through metabolic interactions between catabolic fibroblasts and anabolic cancer cells. This metabolic coupling is induced by ROS-induced metabolic stress responses, resulting in heightened oxidative stress, autophagy, glycolysis, and senescence in fibroblasts.^124,125^ Senescent fibroblasts, including CAFs, undergo substantial metabolic changes, facilitating the export of high-energy metabolites such as lactic acid, ketone bodies, fatty acids, and glutamine through monocarboxylic acid transporter 4 (MCT4). These exported metabolites serve as essential energy sources for cancer cells.^126–128^ Furthermore, mitochondrial dysfunction in senescent fibroblasts contributes to the accumulation of nitric oxide, ROS, and other bioactive molecules within the surrounding TME, along with the production of hydrogen peroxide.^126^ Under conditions of oxidative stress, senescent CAFs upregulate glycolytic flux, which not only sustains their own SASP but also alters chemokine signaling—particularly through enhanced CCL2 secretion. This chemokine gradient promotes the recruitment of TAMs, facilitating immune evasion and metastatic niche formation.^129^ Furthermore, CAFs actively remodel their senescent extracellular matrix (ECM) through coordinated activation of glycolysis and glutamine metabolism to regulate non-essential amino acid availability within the tumor ecosystem.^130^ This cascade fosters a positive feedback loop between ROS release and cell senescence, thereby perpetuating a distinctive metabolic microenvironment conducive to cell senescence and enhanced cancer aggressiveness.

In contrast, impaired metabolism has been noted in T cells with irreversible proliferation stagnation following low-dose IR exposure, which is detrimental for T cell activation. This phenotype manifests as a notable reduction in glucose uptake, glycolysis, and metabolic efficiency.^104^ As CD8 + T cells rely on glycolysis during terminal differentiation,^131^ these alterations underscore the potential consequences of TIS-induced metabolic dysfunction on the antitumor function of T cells. This scenario suggests a plausible therapeutic vulnerability leading to immune evasion by the majority of cancer cells. Evidence supporting this hypothesis includes the observation of anergy in NK cells, weakening their anticancer function following intense stimulation by cancer therapy.^132^ Furthermore, lactate-driven M2 polarization of TAMs enhances pituitary adenoma invasiveness via SASP-mediated CCL17 secretion.^133^ Consequently, metabolic dysfunction in senescent immune cells may diminish their ability to compete for nutrients or energy with cancer cells, thereby fostering hypermetabolic reactions in cancer cells.

### Immune dysfunction and evasion

Following anticancer therapy, senescent cells can elicit a potent immunological response, potentially reshaping the immune TME and enabling evasion of immune surveillance.^134^ Immunological dysfunction is directly associated with TISnt immune cells. As discussed above, when immune cells undergo TIS, they may lose their normal function and become ineffective in carrying out their roles in immune surveillance and defense against cancer cells. This dysfunction could impair the ability of the immune system to recognize and eliminate cancer cells, potentially leading to treatment resistance and disease progression.

The extrinsic impact of neighboring senescent cells holds promise for modulating the immune TME. SASP activation, plays pivotal roles in perturbing the surrounding TME.^135^ It initially aids in the clearance of senescent cells by the innate immune system.^136^ However, heightened SASP production by these senescent cells can create a permissive microenvironment for immune evasion by TISnt cancer cells.^137^ In a murine lipid metabolism model, p53 accumulation triggered hepatocellular carcinoma (HCC) -associated SASP inflammatory responses—including upregulation of hepatocyte growth factor (HGF), TNF, IL-1β, and CCL2—culminating in an immunosuppressive TME conducive to HCC progression.^138^ Emerging evidence highlights the pivotal role of TISnt cells in reprogramming TAMs toward a pro-tumorigenic M2 phenotype through distinct secretory mechanisms. IR-induced senescent lung cancer cells can polarize macrophages towards the M2 phenotype, and this polarization is dependent on IL-13, regulated by the IGF-1 signaling pathway.^139^ In chemotherapy-induced senescent colon cancer cells, the elevated M-CSF expression promotes the polarization of macrophages from the M1 to M2 phenotype.^140^ Additionally, TISnt cancer cells are characterized by their hypermetabolic nature, leading to increased consumption of glucose and amino acids. This metabolic behavior establishes conditions of nutrient deprivation, impairing T cell metabolism and consequently compromising immune cell function.^141,142^ Notably, in patients with refractory melanoma treated with adoptive T cell therapy, tumor cells exhibit heightened glycolytic activity,^143^ indicating a potential mechanism following anticancer therapy through which cancer cells exploit T cell energy resources, leading to functional impairment.

The dispersion and interactions of stromal cells around immune cells in the TME are also crucial determinants of immune cell function and ultimately influence the efficacy of immune-mediated clearance of cancer cells. As discussed, the accumulation of senescent stromal cells can lead to the reconstruction of inflammatory microenvironments.^18,144^ Within such environments, the functionality of CD8 + T cells can be hindered. This impairment enables cancer cells to evade immune surveillance. Senescent stromal cells from aging human skin exhibit a robust SASP characterized by the release of canonical proinflammatory factors. This SASP contributes to the development of an immunosuppressive microenvironment by increasing the presence of MDSCs and Treg cells, which also inhibits the function of CD8 + T cells.^19^ CDK4/6 inhibitors in melanoma cause a potent SASP of TISnt fibroblasts, leading to the recruitment of Gr-1-positive immune cells that suppress antitumor immune response, thereby facilitating melanoma growth.^115^ Furthermore, irradiation triggered the expression of atypical MHC molecules, such as MICA/B and HLA-E, in senescent populations of both dermal fibroblasts and umbilical vein endothelial cells. These molecules interact with NKG2A expressed by NK and CD8 + T cells. This interaction suppresses immune responses to senescent cells.^89^

Clearly, immune dysfunction triggered by senescent cells can create a feedback loop that promotes the accumulation of TIS and the development of an immunosuppressive microenvironment. This condition, in turn, supports the immune escape of cancer cells, fostering their cancer recurrence or metastasis. Although the precise mechanisms underlying immune dysfunction and cancer cell evasion following cancer therapy remain unclear, their detrimental impact on cancer treatment warrants attention.

### Senescence escape and stemness reprogramming

TIS can halt the proliferation of oncogenic cells.^47^ However, given their genomic instability, some cancer cells can develop mechanisms to bypass senescence, allowing them to evade the growth arrest induced by therapy. As reported, a small subset (1/106) of H1299 lung cancer cells lacking p16^INK4a^ and p53 evades senescence induced by camptothecin and restores proliferation.^48^ Among adriamycin-induced senescent MCF-7 cells, a type of breast cancer cell with wild-type p53 and null for p16^INK4a^, a smaller population (1/27 000) managed to evade persistent growth arrest, potentially acquiring resistance to therapies that induce senescence.^145^ Subsequent research has further confirmed that cancer cells that evade the senescent state induced by chemotherapy or radiation therapy.^51,146^ This evasion mechanism reduces the effectiveness of treatment and increases the risk of cancer recurrence, highlighting the importance of understanding and addressing this aspect of cancer biology.^134,147^

Emerging evidence highlights a paradoxical link between TIS and CSC generation, where senescent cancer cells can acquire stem-like properties to evade growth arrest and drive tumor recurrence. In prostate cancer, doxorubicin-induced senescent cells exhibit enhanced survival through CD133 + /CD44+ stemness markers, suggesting senescence escape via stemness reprogramming.^148^ Similarly in acute lymphoblastic and acute myeloid leukemia models,^5^ senescent lymphoma cells targeting H3K9me3 or p53 have been shown to spontaneously escape adriamycin-induced TIS. Upon escaping senescence, these cells undergo reprogramming into CSCs capable of initiating leukemia, exhibiting heightened self-renewal ability and invasiveness dependent on the Wnt signaling pathway.^5^ Furthermore, breast cancer cells that evade TIS exhibit a flexible cancer stem cell-like identity, and distinct metabolic traits rely on glutamine.^122^ Mechanistically, epigenetic regulation-including DNA methyltransferases (Dnmt1, Dnmt3A), TET enzymes (Tet1, Tet3), and chromatin remodelers (Jmjd3, Utx)- drive this senescence-stemness reprogramming, enabling cancer cells to bypass senescence and acquire stemness.^149^ These findings also indicate that the stemness reprogramming of TISnt cancer cells may serve as a potential origin of CSCs.

This phenomenon, termed “senescence-associated stemness”,^150^ is further exemplified in liver cancer, where non-stem (EpCAM-/CD133-) cells transition into Wnt/β-catenin-activated CSCs post-senescence, enhancing tumor initiation.^66^ Another study^151^ showed that some senescent breast cancer cells are able to escape senescence and establish stable colonies with a CSC-like aggressive phenotype characterized by elevated CD133 and Oct4 expression. This phenomenon was also observed in human breast tumors following neoadjuvant chemotherapy.^151^ Additional studies revealed a novel epigenetic synergism between the histone variant macroH2A1 and DNA methylation in hepatocellular carcinoma (HCC), facilitating the escape of cancer cells from drug-induced senescence, thereby promoting the development of a senescent-like phenotype.^152^ Hypoxia further reinforces this link by modulating the HIF-1α-TWIST-p21 axis to induced senescence but sustain a pro-oncogenic CSC phenotype.^153^ In head and neck cancer,^154^ Pot1b loss triggers senescence, suppressing tumorigenesis in p53 + /+ mice but failing to block Lgr6+ CSC expansion in p53−/− tumors, leading to metastasis. Depleting K15+ stem cells or blocking Cxcr2/PI3K signaling reduces CSCs and induces senescence, underscoring the dual role of senescence in both tumor suppression and CSC-driven relapse.

However, further studies are needed to determine whether other stromal cell types in the TME also have the potential to evade senescence as well as stemness reprogramming. Indeed, induced pluripotent stem cells (iPSCs) are established by introducing specific transcription factors into somatic cells such as fibroblasts. These factors reprogram the cells to a pluripotent state. Research has shown that SASP components, such as IL-6, can enhance the cellular reprogramming of iPSC generation.^155^ This discovery raises the possibility that certain senescent stromal cells within the senescent microenvironment may also engage in specific reprogramming mechanisms to evade senescence and obtain stemness.

### An accumulated senescent state

**Fig. 3:**
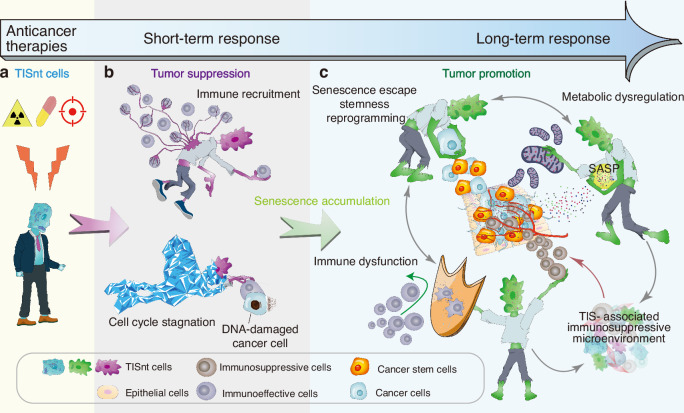
A TIS-associated immunosuppressive microenvironment orchestrated by accumulated TISnt cells. **a** Anticancer therapy induces TISnt cells. **b** In the early stages of cancer treatment, a tumor-suppressive effect is exerted by senescent cancer cells due to their arrested proliferation and ability to recruit immune cells. **c** As senescent cells persist within the TME for a long time, a persistently accumulating immunosuppressive senescent state can be formed through multifaceted and interactive mechanisms, including SASP, metabolic dysregulation, senescence escape and stemness reprogramming, and immune dysfunction. *Created with Adobe Illustrator Artwork*

The accumulation of age-related senescent cells within tissues or organs can lead to deleterious consequences and the establishment of a protumorigenic environment.^30,156^ TISnt cells, characterized by their enduring viability within the TME, exhibit traits of cellular rigidity and immortality attributable to irreversible cell cycle arrest. The accumulation of TISnt cells within the TME orchestrates a senescence-amplified condition characterized by chronic inflammatory and immunosuppressive attributes (Fig. 3). Additionally, while a minority of TISnt cancer cells manage to circumvent senescence, they have the capacity to undergo reprogramming toward a CSC phenotype. The presence of senescent reprogrammed CSCs exacerbates the cellular heterogeneity within the senescent microenvironment. These cells disrupt the immunosurveillance mechanisms of the immune microenvironment, thereby impeding the effective elimination of cancer cells, including CSCs, to some degree.^157^

Stromal TISnt cells can maintain a long-term cell cycle arrest state. For instance, lung fibroblasts induced to senesce by fractionated 48 Gy irradiation have been shown to survive for more than 6 months.^75^ A murine model engineered to accumulate senescent fibroblasts through activation of the p27Kip1 allele has been established, revealing the establishment of an inflammatory microenvironment reliant on increased IL-6 levels, subsequently fostering tumor growth.^158^ The accumulation of senescent cells has been associated with an increase in the stromal SASP.^135^ The stromal senescence-derived SASP further causes an increase in immunosuppressive cellular subsets, such as Treg cells, along with associated signaling molecules, culminating in the inhibition of CD8 + T cell functionality. This immunosuppressive microenvironment confers protection to cancer cells against immune-mediated cytotoxicity.^19^ Senescent endothelial cells induced by sunitinib chemotherapy exhibit prolonged survival and significant accumulation.^93^ This TIS-associated accumulation contributes to the secretion of SASP factors, which can attract breast cancer cells to migrate toward blood vessels. Concurrently, the durative downregulation of vascular endothelial cadherin (VEC) in senescent endothelial cells may exacerbate the permeability of the vascular endothelium, further enhancing the penetration and dissemination of cancer cells.

Overall, the intricate secretory profiles of senescent cells posttreatment underscore the significant contribution of the SASP to cancer progression and treatment response. The SASP factors also play a pivotal role in fostering the progression of senescence within neighboring cells, thereby establishing a self-perpetuating cycle culminating in the accumulation of senescent cells. Concurrently, inflammatory mediators, prominently interleukins and chemokines constituting key constituents of the SASP, exert pronounced effects on immune cell recruitment, particularly favoring the influx of immunosuppressive cell populations (Fig. 3). A persistent inflammatory state evokes compensatory immunosuppression, which inhibits proinflammatory processes by impairing the functions of effector immune cells,^159^ e.g., macrophages, T cells and NK cells. Unfortunately, these cells are indispensable for immune surveillance and the subsequent clearance of senescent cells^160^; i.e., inflammation-induced immunosuppression prevents the clearance of host tissues. The orchestrated interplay between the SASP, chronic inflammation and immunosuppression, emerges as a significant contributor to an increased senescence state.

## Targeting therapy-induced senescent cells in cancer: opportunities and challenges

**Fig. 4:**
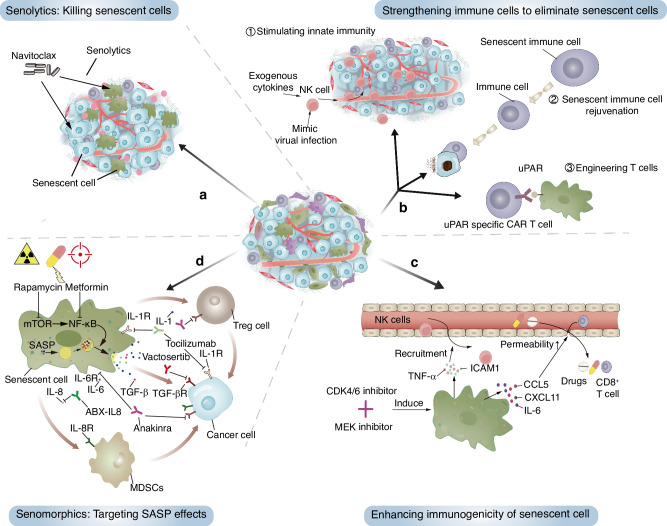
Current strategies targeting TISnt cells in cancer. **a** Senolytics such as navitoclax can be used to kill senescent cells directly. **b** Strengthening immune effector cells to senescent cells, including stimulating innate immunity, rejuvenating senescent immune cells, and engineering senescence-specific CAR-T cells. **c** Enhancing the immunogenicity of senescent cells to recruit effector immune cells, such as by augmenting chemotactic and inflammatory SASP factors. **d** Targeting the SASP to eliminate its tumor-promoting effect by blocking pathways related to the SASP or focusing on a single crucial SASP component. *Created with Adobe Illustrator Artwork*

Despite the detrimental effects of TISnt cells, they also represent a potential focal point for improving cancer therapy efficacy. Efforts to develop effective strategies targeting TISnt cells have gained traction in recent years. These approaches aim to clear TISnt cells or inhibit their function to improve cancer therapy (summarized in Fig. 4 and listed in Table 2). Combining such targeted approaches with conventional treatments represents a synergistic approach, namely a one-two punch therapy, to address the challenges posed by TISnt cells.^161^

**Table 2:** Strategies targeting TIS have the potential as the second punch to anticancer therapy

Compounds/Strategies		Phase; Status	Registration number	Targets	Confirmed benefits against cancer	Refs
**Senolytics**	Dasatinib and Quercetin	Phase II; Recruiting	NCT06355037	Panreceptor tyrosine kinases	Reducing the burden from senescent cells in radiation-exposed mice	^243^
	Navitoclax (ABT-263)	Phase II; Completed	NCT02591095	Pro-survival proteins (BCL-2/Bcl-xL)	Reducing cancer recurrence in doxorubicin - treated mice; restraining the recurrence of gliomas in mice	^76,166^
	Venetoclax (ABT-199)	Phase III; Enrolling by invitation	NCT06544109	Pro-survival proteins (BCL-2/Bcl-xL)	Increasing cell death in irradiated sarcoma cells	^244^
	Cardiac glycosides	Early phase I; Completed	NCT03928210	BCL-2, Na + /K+ ATPase pump	Killing tumor cells, eliminating IR-induced senescent cells and senescent preneoplastic cells	^245,246^
	Sertraline	Phase I; Completed	NCT02891278	mTOR	Inducing apoptosis in XL413-induced senescent liver cancer cells	^57^
	MCL-1 inhibitor	Phase I; Completed	NCT02992483	Anti-apoptotic protein MCL-1	Eliminating senescent tumor cells and metastases in tumor bearing mice	^247^
	CDK4/6 inhibitor combined with MEK inhibitor	Phase I; Unknown	NCT04615312	Immune escape	Stimulating the NK cell surveillance program, promoting tumor infiltration by CD8 + T cells and promoting tumor regression	^53,190^
	GL-V9	Preclinical animal models		Lysosomes, mitochondria, ROS	Eradicating senescent embryonic fibroblasts and senescent breast cancer cells induced by doxorubicin	^167^
	FOXO4-p53 peptides	Preclinical animal models		FOXO4-p53	Eliminating IR-induced senescent fibroblasts and radiosensitizing non-small cell lung cancer cells	^168^
	Piperlongumine	Preclinical animal models		Oxidation resistance 1 (OXR1); NF-κB	Killing IR-induced senescent human WI-38 fibroblasts	^248^
	Galactose-modified duocarmycin	Preclinical animal models		SA-β-Gal	Selectively reducing the number of β-catenin-positive preneoplastic senescent cells	^173^
	Galactose modified senolytic (Nav-Gal)	Preclinical animal models		SA-β-Gal and pro-survival proteins	Eradicating senescent lung cancer cells in vivo by combination therapy, less side effects	^172,174^
	Senolytic CAR T cells	Preclinical animal models		uPAR	Prolonging the survival of senescence-inducing drug-treated lung adenocarcinoma mice	^181^
**Senomorphics**	Anakinra	Phase II; Recruiting	NCT06710197	Self-amplifying feedback loop of SASP (IL-1R)	Preventing senescence of inflammatory CAF, sensitizing mice bearing rectal cancer to irradiation	^196^
	Tocilizumab	Phase IV; Recruiting	NCT05855122	Self-amplifying feedback loop of SASP (IL-6R)	Preventing PGCC formation, attenuating embryonic stemness and the CAF phenotype, and inhibiting tumor growth	^200^
	Vactosertib	Phase I; Completed	NCT03143985	TGF-βR	Eliminating senescent cells, reducing SASP levels, diminishing immunosuppressive immune cell infiltration	^26^
	Rapamycin	Phase I; Not yet recruiting	NCT06743503	mTOR	Inhibiting SASP, limiting the tumor-promoting ability of senescent mouse fibroblasts in vivo	^205^
	Metformin	Phase II; Completed	NCT03053544	NF-κB	Inhibiting SASP, blocking cancer metabolic plasticity and growth	^207,208^
	Tazemetostat	Phase II; Not yet recruiting	NCT06068881	EZH2	Promoting the production of SASP chemokines (CCL2 and CXCL9/10) to increase NK and T cells infiltration	^209^
	MitoQ	Not Applicable; Unknown	NCT05146843	Mitochondria	Inhibiting the SASP, reducing mitochondrial ROS production	^210^
	FK866	Phase III; Completed	NCT03674294	NAMPT	Suppressing SASP production, preventing senescent cells from stimulating cancer cell proliferation	^118^
	Trabectedin	Phase II clinical trial; Unknown	NCT05131386	Glutamine metabolism	Suppressing cancer stem-like cells escape from TIS	^211,212^

### Senolytics to directly eliminate senescent cells

Killing senescent cells using pharmacological compounds, called “senolytics”, is the most common strategy for clearing senescent cells. Compared with nonsenescent cells, TISnt cells with DNA damage are considered to be more likely to undergo apoptosis; however, increased resistance to apoptosis enables them to survive.^162,163^ Multiple pro-survival proteins and pathways have been explored as potential targets of senolytics (Table 2), most of which have shown promising results in preclinical trials.

For instance, one of the most studied senolytics is Navitoclax (ABT-263),^164^ a BH3 mimetic targeting the antiapoptotic proteins BCL2/BCL-XL/BCL-W, which has been confirmed to effectively eliminate TISnt cancer cells in doxorubicin-treated mice exhibiting breast cancer^76,165^ and radiotherapy-treated mice exhibiting glioma.^166^ These preclinical studies showed that senolytics kill TISnt cancer cells by inhibiting pro-survival signals, which can also reduce tumor recurrence. Several natural products and their derivatives exhibit potential as senolytic agents capable of eliminating TISnt cancer cells and thereby enhancing cancer therapy outcomes.^144^ GL-V9, a synthetic flavonoid analog of wogonin, has been shown to eradicate senescent breast cancer cells induced by doxorubicin, as well as senescent mouse embryonic fibroblasts.^167^ Indeed, senolytic agents designed to target senescent cells within the TME have shown promise in mitigating the adverse effects associated with TIS. FOXO4-p53 peptides have demonstrated efficacy in eliminating IR-induced senescent fibroblasts. Moreover, these peptides have been found to sensitize non-small cell lung cancer cells to radiotherapy, potentially enhancing treatment outcomes.^168^ However, senolytics do not display high enough specificity to distinguish senescent cells from normal cells, which may lead to secondary defects such as neutropenia and thrombocytopenia,^169,170^ limiting the clinical application of senolytic therapies in human diseases.

A new senolytic strategy has been designed by exploiting the SA-β-gal activity of senescent cells to enhance their sensitivity to target senescent cells.^171^ Cytotoxic drugs coated with galactooligosaccharides have been preferentially delivered to senescent cells, achieving specific killing of TISnt cancer cells after chemotherapy.^172^ Galactose-modified duocarmycin (GMD) enhances the clearance of senescent cells that accumulate after total body irradiation in mice, and the administration of GMD has been shown to selectively reduce the number of β-catenin-positive preneoplastic senescent cells in adamantinomatous craniopharyngioma (ACP) mouse models.^173^ A recent study also revealed that a potent senolytic prodrug (Nav-Gal), which is a combination of ABT-263 and galactose, significantly eradicates TISnt lung cancer cells in vivo when combined with cisplatin treatment, suppressing tumor growth and ABT-263-induced platelet apoptosis.^174^ Treatment strategies based on the characteristics of senescent cells have great potential to improve the specificity for killing TISnt cells, which contribute to improved cancer treatment outcomes.

### Strengthening immune cells to eliminate senescent cells

Mobilizing and enhancing the anticancer immunity of the TME and further controlling and killing cancer cells has been proposed as one of the most promising research directions in the field of cancer therapy. A senescent TME with TISnt cells accumulated develops during or after cancer therapy, resulting in an immunosuppressive state.^19,89^ Therefore, restoring a functional immune microenvironment might be another effective approach for eliminating TISnt cells.^175^

Enhancing the activity of immune cells or rejuvenating the senescent immune system is one approach that has been used to assist in the surveillance of senescent cancer cells. The phenomenon of T cell dysfunction resulting from both senescence and exhaustion poses a significant limitation to the efficacy of anticancer immunotherapy. One proposed strategy to mitigate this limitation involves preventing senescence in tumor-specific T cells through the inhibition of ATM and/or MAPK signaling pathways. Concurrently, combining with anti-PD-L1 checkpoint blockade is suggested to synergistically enhance antitumor immune responses and thereby improve the overall efficacy of immunotherapy.^176^ IL-15 can regulate the delay or reversal of senescence in CD8 + T cells through activation of the JAK3-STAT5 signaling pathway. This mechanism holds promise for rejuvenating immune cells, thereby enabling them to mount effective antitumor responses, particularly in the context of TIS.^177,178^ As reported,^179^ the combined treatment regimen involving a PARP inhibitor and a CDK4/6 inhibitor effectively restructured an antitumor environment characterized by heightened infiltration of CD8 + T cells and NK cells, as well as a diminished presence of macrophages and granulocytic MDSCs. Further complementing this regimen with an anti-PD-L1 approach can target TISnt cells triggered by these combined therapies. This one-two punch approach reveals synergistic antitumor and immunomodulatory effects, thereby augmenting therapeutic efficacy.

Updated protein and cell engineering strategies have made great strides in enhancing the antitumor function of Engineered tumor-specific chimeric antigen receptor (CAR) T cells, increasing cancer-targeting specificity, reforming the TME to improve immunotherapeutic outcomes, and preventing tumor escape and relapse.^180^ CAR-T cells also serve as effective senolytic agents for targeting senescent cells.^181^ In one study, CAR-T cells were constructed specifically targeting urokinase plasminogen activator receptor (uPAR), a cell surface protein widely expressed during senescence. These uPAR-specific CAR-T cells effectively killed senescent cells, prolonged the survival of mice with lung adenocarcinoma treated with chemotherapy, and restored tissue homeostasis to protect mice from liver fibrosis.^181^ Moreover, CAR-T cells that target uPAR have been shown to have good therapeutic effects on ovarian cancer.^182^ However, a great challenge in using CAR-T cells to eliminate senescent cells exists; this strategy is highly dependent on the recognition of specific senescent cell markers that are abundant and expressed at different levels in cells of different origins.^183^ Additionally, the potential cytokine storm induced by CAR-T cell therapy requires further investigation.^184^

Furthermore, Polyinosinic-polycytidylic acid, which mimics viral infection, was administered to stimulate the innate immune response and was shown to enhance the cytotoxicity of NK cells in the liver to clear senescent cells, further controlling liver fibrosis.^185^ Although this drug has not been considered for clinical use due to possible side effects, this result indicated that NK cell activation may be an effective senolytic strategy. This strategy is also supported by studies showing that IL-15 and IL-21, which are SASP compounds, significantly promote NK cell-mediated antitumor effects.^186,187^ Chaturvedi et al. made a significant discovery suggesting that the heterodimeric bifunctional fusion protein complex HCW9218, which combines IL-15 immunostimulatory and TGF-β antagonist activities, stimulated NK cells to reduce TISnt cells and decreased SASP factors in nontarget tissues affected by chemotherapy in mice with tumors.^188^ However, in a senescent TME, whether senescent NK cells are stimulated or cleared and how senescent immune cells regulate tumor survival are more complex remaining further exploration.

### Enhancing the immunogenicity/antigenicity of senescent cells

The immunogenicity or antigenicity of cancer cells, which refers to their capacity to elicit an immune response, plays a pivotal role in activating specific immune cells, thereby stimulating their proliferation and differentiation. This pivotal process dictates the efficacy of immune surveillance in recognizing, monitoring, and eradicating cancerous cells. Consequently, an alternative strategy to target TISnt cells involves augmenting their immunogenicity, thereby facilitating the recruitment of a greater number of functional immune cells. Notably, the robust inflammatory profile exhibited by senescent cells, reminiscent of cancer antigens, represents one avenue through which this enhancement can be achieved.

For instance, the coadministration of a CDK4/6 inhibitor with trametinib yielded a more pronounced TIS state with proliferation arrest in KRAS-mutant lung cancer cells. This combination therapy amplified the secretion of SASP components such as TNF-α and intercellular adhesion molecule-1 (ICAM-1). This heightened expression of SASP factors further bolstered the surveillance of NK cells, culminating in enhanced tumor regression.^53^ CCL2, another prevalent component of the SASP, has been shown to play a role in the recruitment of NK cells. This recruitment mechanism has been shown to effectively eradicate senescent liver cancer cells in murine models.^189^ The concurrent administration of CDK4/6 and MEK inhibitors in pancreatic ductal adenocarcinoma (PDAC) has been shown to elevate the levels of vascular endothelial growth factor (VEGF), leading to enhanced vascular density. However, this treatment regimen also induces the secretion of proinflammatory SASP molecules such as IL-6, CCL5, and CXCL1, which in turn promote senescence in endothelial cells, thereby increasing vascular permeability. Consequently, this creates a favorable microenvironment for the infiltration of CD8 + T cells into the TME and facilitates the diffusion of the chemotherapy drug gemcitabine, resulting in the effective elimination of cancer cells.^190^ Furthermore, the modified SASP resulting from autophagy inhibition exhibited heightened efficacy in inducing paracrine senescence in neighboring glioblastoma multiforme (GBM) cells. It promoted a shift in macrophage polarization toward the antitumor M1 state and hindered the recruitment of protumor neutrophils to GBM tumor tissues.^191^

However, hastening the production of SASP may also exacerbate the senescent phenotype, potentially perpetuating the remodeling of the senescent microenvironment. This phenomenon, in turn, is counterproductive for efficacious cancer treatment. This also highlights the dynamic nature of the SASP, which can shift depending on factors such as the stage of senescence, the presence of other cell types in the microenvironment, and the overall context of tissue physiology and pathology.^27,192^ Therefore, the development of methodologies capable of precisely regulating the equilibrium of the SASP within the senescent TME presents a significant hurdle in realizing the optimal outcome of targeting senescent cells to augment cancer therapy.

As mentioned above, senescent cells can be targeted for elimination directly through the use of senolytics or indirectly through immune-mediated mechanisms. However, a major challenge in clearing senescent cells lies in the low specificity of senolytic agents observed in preclinical studies. Improving specificity is crucial, as complete eradication of senescent cells may introduce novel complexities to cancer therapy outcomes and patient overall health. The comprehensive removal of senescent cells may have adverse effects on tissue repair processes, as evidenced by studies demonstrating wound healing defects in mice subjected to senescent cell clearance.^193^ Notably, recent research has highlighted the deleterious consequences of eliminating senescent hepatic sinusoidal endothelial cells in murine models. This intervention results in the disruption of blood-tissue barriers, exacerbating liver fibrosis and compromising overall health. Importantly, the absence of replacement for eliminated senescent sinusoidal endothelial cells underscores the structural and functional deterioration observed.^194^ These findings suggest that elderly cancer patients receiving treatments aimed at eliminating TISnt cells may face heightened health risks, as their physiological resilience to such interventions may be compromised.

### Targeting the SASP of therapy-induced senescent cells

The potential risks associated with the direct elimination of senescent cells have prompted scholars to shift their focus toward the senescence-associated secretory phenotype (SASP).^195^ In combination with predominantly proinflammatory and immunosuppressive factors, the SASP is a promising target for intervention. Consequently, strategies aimed at modulating the SASP, termed senomorphics (Table 2), have been developed. Unlike senolytics, senomorphic drugs do not induce cell death but rather aim to mitigate the detrimental effects of the SASP. According to the targets of the SASP, senomorphics can be categorized into four main groups: those aimed at inhibiting individual SASP factors, those targeting key signaling molecules involved in SASP regulation, those modulating SASP-related metabolic vulnerabilities, and those SASP-regulated immune modulation.

Among SASP cytokines, both IL-1 and IL-6 are proinflammatory cytokines. In the context of senescence, IL-1 and IL-6 contribute to the establishment and maintenance of the senescent phenotype by promoting inflammation and reinforcing senescence-associated growth arrest.^196–199^ Anakinra and tocilizumab, which individually antagonize IL-1R and IL-6R, effectively block the self-amplifying feedback loop of IL-1 and IL-6, thereby impeding the senescence process.^108^ A study utilizing a patient-derived xenograft high-grade serous ovarian carcinoma model demonstrated that the inhibition of the IL-6-mediated SASP feedback loop can prevent PGCC formation, attenuate embryonic stemness, and suppress the CAF phenotype, ultimately relieving cancer progression and sensitizing tumors to chemotherapy.^200^ Similarly, IL-1 inhibition, an approach contributing to the prevention of inflammatory CAF senescence in radiotherapy, could sensitize mice bearing rectal cancer to irradiation.^196^ Like IL-6 and IL-1, TGF-β is one of the most prominent SASP factors associated with chronic inflammation and immune suppression in cancer.^201–203^ As reported,^204^ cellular senescence can spread between hepatocytes in a loop driven by TGF-β released from macrophages. Inhibiting TGF-β signaling following acetaminophen-induced liver injury decreased senescence, leading to enhanced regeneration and increased survival in a mouse model of acute liver injury and failure. Furthermore, in an immunocompetent mouse model of lung cancer, TGF-β induced a more profound senescent state under hypoxic conditions, and blocking TGF-β signaling in tumors eliminated naturally occurring senescent cells, reduced SASP levels, and diminished immunosuppressive immune cell infiltration, ultimately leading to antitumor effects.^26^

Another effective method of senomorphics involves blocking the key signaling pathways regulating SASP to attenuate the negative effect on cancer therapy. For example, mTOR is the key molecule that mediates SASP pathways by regulating the translation of upstream regulatory factors, such as the NF-κB and MK2 kinases.^205,206^ Some scholars have reported that rapamycin, an mTOR inhibitor, selectively inhibits the SASP effect in senescent mouse fibroblasts, limiting the tumor-promoting ability of the SASP in vivo.^205^ Metformin, which blocks cancer metabolic plasticity and growth,^207^ reduces SASP production by regulating NF-κB signaling in aging-related diseases.^208^ The TIS-induced reshaped TME in pancreatic ductal adenocarcinoma (PDAC) suppresses the surveillance functions of NK cells and T cells by epigenetically repressing proinflammatory SASP genes, a process facilitated by EZH2 activity. Consequently, the blockade of EZH2 has been demonstrated to promote the production of SASP chemokines such as CCL2 and CXCL9/10. This augmentation leads to increased infiltration of NK cells and T cells into the TME, ultimately resulting in the eradication of PDAC in murine models.^209^

A sophisticated interplay between the SASP and cellular metabolism has been revealed, suggesting the potential to disrupt SASP effects by modulating cancer metabolism. Treatment with the mitochondria-targeting antioxidant MitoQ inhibits the SASP in senescent melanocytes, thereby reducing the associated epidermal damage. This treatment further mirrored the effects of the senolytic drug ABT737 in eliminating senescent melanocytes.^210^ Upregulated expression of nicotinamide phosphoribosyltransferase (NAMPT) in senescent cells enhances glycolytic and mitochondrial oxidative phosphorylation activities via NAD+ metabolism. Inhibiting NAMPT suppresses SASP production through AMPK-p53 signaling, preventing senescent cells from stimulating cancer cell proliferation.^118^ Cell survival after irradiation hinges on boosting glutaminolysis-dependent energy metabolism.^211^ Inhibiting glutaminolysis led to the emergence of β-galactosidase-positive and IL-6/IL-8-secreting cells within irradiated tumor cells. When ABT263 was administered in combination, these senescent cells transitioned into apoptotic cells. These results reinforce the idea that combining a glutaminolysis inhibitor with a senolytic agent can significantly improve the effectiveness of radiotherapy.^212^ In addition, Lactate fosters a pro-inflammatory and pathogenic phenotype in aged B cells, contributing to the release of SASP mediators and autoimmune antibodies such as anti-dsDNA. Mediated by SLC5A12, these lactate-driven effects also shift CD4 + T cells toward a pro-inflammatory state. Targeting lactate metabolism in B cells presents a potential strategy to reduce inflammation and senescence, thereby mitigating immune dysfunction in cancer.^213,214^ Furthermore, inhibiting PDK4 redirects cellular metabolism toward mitochondrial oxidative phosphorylation, curbing SASP-associated cytokine production and enhancing the sensitivity of senescent cells to apoptosis-inducing agents. Combining lactate export inhibition and PDK4 targeting with senolytic drugs may enhance therapeutic efficacy in addressing TIS and improving cancer treatment outcomes.^123,215^

The interplay between SASP modulation and immune therapy warrants further exploration. Some SASP factors, such as chemokines like CCL2 and CXCL10, can recruit cytotoxic T cells and NK cells, potentially enhancing the immune response against tumors. Conversely, other SASP components, including TGF-β and IL-10, contribute to immunosuppression by fostering regulatory T cells (Tregs) and M2 macrophages.^216,217^ Selectively targeting the immunosuppressive elements of the SASP while preserving its immune-stimulatory effects could enhance the efficacy of immune checkpoint inhibitors (ICIs) and restore anti-tumor immunity. A study demonstrated that enhancing the SASP in senescent ovarian cancer cells using topoisomerase 1 (TOP1) inhibitors like irinotecan sensitized tumors to anti-PD-1 therapy.^218^ This approach resulted in elevated infiltration of activated CD8 + T cells and dendritic cells into the tumor bed, leading to reduced tumor burden and improved survival in a syngeneic mouse model. TGF-β, a key SASP component, exerts potent immunosuppressive effects within the TME. By inhibiting CD8 + T cell activity, TGF-β facilitates lung metastasis of HCC. However, the combination of a TGFβR1 inhibitor with anti-PD-L1 therapy significantly suppressed SOX18-mediated HCC progression and metastasis, demonstrating a strong therapeutic synergy.^219^ Furthermore, SASP-responsive macrophages often adopt an M2-like phenotype, contributing to tumor progression. Inhibiting the colony-stimulating factor 1 receptor (CSF1R) can repolarize macrophages towards a pro-inflammatory M1-like phenotype, enhancing anti-tumor immunity and counteracting the tumor-supportive effects of the SASP. A study revealed that CSF1R inhibitors facilitated macrophage repolarization by blocking cholesterol biosynthesis, which significantly increased the efficacy of PD-1 antibody treatment in colorectal cancer models.^220^ These findings underscore the potential of combining SASP-modulating therapies with ICIs to enhance anti-tumor immune responses. While direct evidence of SASP-targeting strategies in combination with ICIs remains limited, these preclinical studies provide a solid foundation for further research and the development of more effective cancer immunotherapies.

Overall, the involvement of the SASP in anticancer therapy is highly intricate, suggesting that indiscriminate targeting of the SASP could lead to unintended consequences, such as impeding immunological surveillance. Studies have shown that inhibiting IL-1 signaling can disrupt immune surveillance, facilitating cancer cell evasion and survival in the presence of therapies.^22^ Conversely, elevating certain SASP components has been found to enhance the recruitment of immune cells, promoting the elimination of TISnt cancer cells.^190^ Therefore, it is crucial to carefully modulate SASP components within the TME by specifically targeting identified markers rather than arbitrarily reducing them.

## Conclusions and perspectives in the CONTEXT of head and neck cancer

TIS is a common response to cancer therapies, but accumulating evidence shows that TISnt cells persist in the tumor microenvironment (TME) and contribute to immunosuppression, hindering treatment effectiveness. These heterogeneous TISnt cells, particularly those expressing SASP factors, create a chronic inflammatory environment that promotes tumor progression and therapy resistance. Metabolically dysregulated senescent cells may further suppress immunity by interacting with proinflammatory SASP components, while also evading immune surveillance through immune checkpoint molecules, such as PD-L1. Additionally, senescent cells undergoing stemness reprogramming gain resistance to apoptosis and contribute to a more heterogeneous, immune-evasive microenvironment. To address this, targeting TISnt cells with senolytics, combined with strategies to modulate the SASP or enhance immune responses, may help reduce the senescence-accumulated immunosuppression and improve cancer treatment outcomes.

However, navigating the complexities of TISnt cell biology and interactions within the TME remains a formidable task, particularly in the context of head and neck cancer with highly intrinsic heterogeneity. Head and neck cancer is a prevalent malignancy worldwide, with incidence and mortality rates varying across regions due to differences in lifestyle and environmental factors. Despite advances in diagnostic and therapeutic technologies, overall survival rates have seen limited improvement. The primary challenges lie in the high heterogeneity of the disease, late-stage diagnosis in many cases, the immunosuppressive nature of the tumor microenvironment, and the toxicity associated with treatments.^31,221^ Surgical intervention is often limited by the complex anatomy and risk of functional impairment, leaving most patients reliant on radiotherapy, chemotherapy, or targeted therapies.^222^ However, these conventional therapies, while initially effective in reducing tumor burden, are frequently hindered by immune evasion and therapy resistance, and often induce TISnt cells.

As previously reported, resistance to radio- and chemotherapy-induced cell death constitutes a major determinant of treatment failure in oncology^223^ and remains a persistent clinical challenge in head and neck squamous cell carcinoma (HNSCC), which accounts for over 90% of all head and neck cancers.^224^ Scholars revealed that resistance to radiotherapy was strongly associated with the early induction of senescence in HNSCC cells, accompanied by NF-κB-dependent production of senescence-associated cytokines, particularly CXCR2 ligands.^15^ These cytokines, secreted in a non-cell autonomous, paracrine manner, create an immune-evasive microenvironment that fosters radioresistance.^225^ The heterogeneity of HNSCC also influences the composition of SASPs during treatment-induced senescence. Radiotherapy-resistant CAL-33 cells upregulate SASP factors, including IL-1α, IL-1β, CXCL1, CXCL2, CXCL3, CXCL6, CXCL8, GM-CSF, and IL-6. In contrast, the radiotherapy-sensitive UDSCC2 cell line predominantly produces interferon-related SASP molecules such as CXCL10 and CXCL11. Additionally, Cal-27 and HSC6 cells secrete IL-1β, IL-6, CXCL1, GM-CSF, G-CSF, and MMP1, promoting the proliferation of surrounding non-senescent cancer cells through the release of small extracellular vesicles (sEVs).^15,226^ Senescent HNSCC cells regulate cytokine production through the inflammasome/IL-1α/NF-κB axis. Pharmacological inhibition of NF-κB using metformin effectively reduces the production of SASP cytokines, significantly enhancing radiotherapy efficacy both in vitro and in vivo.^227^ Additionally, during cis-diamminedichloroplatinum (II)-induced senescence in OSCC, the TME exhibits increased dense, thick collagen fibers. This desmoplasia-like change is primarily driven by SASP factors, such as CCL5 and IL-13, which induce the transformation of M1 macrophages into an M2 phenotype, further contributing to the immunosuppressive TME.^32^ In light of these findings, the senolytic agent ABT-263 (Navitoclax) has been investigated as a potential therapeutic strategy to improve treatment outcomes in HNSCC, particularly following cisplatin-based chemoradiotherapy.^8^ Furthermore, the combination of CDK4/6 inhibitor palbociclib with ABT-263 has been shown to significantly enhance apoptosis and reduce cell survival in HPV − HNSCC cell lines compared to monotherapy.^9^ In addition, oral fibroblasts undergoing senescence due to cisplatin, other DNA-damaging agents, or aging, as well as by inherently senescent CAFs, contribute to the development of an inflammatory SASP, which promotes the pro-tumorigenic effects of chemotherapy on the TME.^228^ However, while TISnt cancer cells have been well studied in the context of their impact on therapeutic efficacy, there is limited research on the senescence of other cell types within the head and neck cancer microenvironment and their subsequent effects on treatment outcomes.

**Fig. 5:**
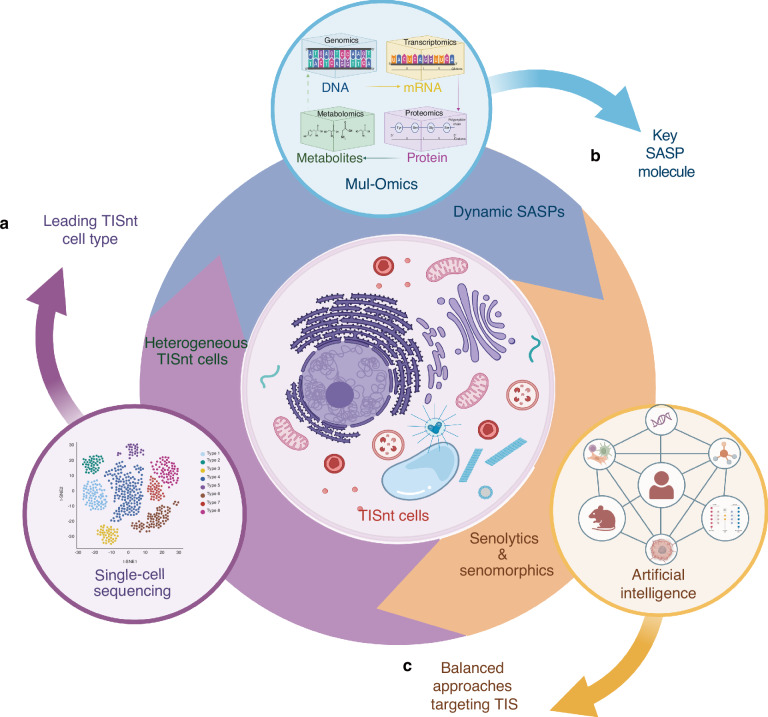
Perspectives on precise and balanced targeting TISnt cells. **a** Given the heterogeneity of TISnt cells within the tumor and its surrounding microenvironment, single-cell sequencing techniques can aid in mapping the senescence-related single-cell transcriptome and identifying the predominant cell type among TISnt cells. This focused approach allows for the precise targeting of the leading cell type driving senescence. **b** The SASP produced by TISnt cells is characterized by their diverse and dynamic nature. To effectively target TISnt cells, it is essential to identify the key SASP factors. This can be achieved through comprehensive omics screening methodologies such as Genomics, RNA-seq, Proteomics, and Metabonomics. By discerning the most critical SASP components, tailored therapeutic strategies can be developed to disrupt the protumorigenic effects of TISnt cells. **c** Senolytics and senomorphics represent promising approaches for targeting TISnt cells. These compounds can be employed in various combinations, taking into account insights into the predominant senescent cell type and the functional composition of the SASP. Additionally, strategies aimed at stimulating host immunity can enhance the efficacy of senolytic and senomorphic therapies, promoting the clearance of TISnt cells by the immune system. *Created with BioRender*

Undoubtedly, the heterogeneity of TISnt cell populations and their dynamic nature underscore the need for comprehensive research efforts to uncover optimal therapeutic targets and strategies. Advances in genetic studies aimed at elucidating the lifecycle of senescent cells hold significant promise for potential breakthroughs. By utilizing single-cell RNA sequencing techniques, researchers have made strides in unraveling the intricate changes in cellular states throughout the lifespan of mice.^229^ This approach has enabled comprehensive mapping of the single-cell transcriptome and cellular specificity across diverse tissues and organs,^230^ facilitating thorough exploration and identification of distinct cell subpopulations implicated in age-related diseases.^231–233^ Single-cell sequencing methodologies offer a valuable tool for generating detailed insights into the landscape of TISnt cells within cancer (Fig. 5a). By characterizing the gene expression profiles and secretome of individual cell types at various stages of senescence, researchers can construct a comprehensive map of TISnt cell dynamics in the context of cancer progression. These techniques, such as the Cellular Senescence Network,^234^ enable the identification of the predominant cell types among TISnt cells, facilitating a focused approach that allows for the precise targeting of the specific cell types driving senescence within TME. This holistic understanding of TISnt cells, encompassing their molecular signatures and functional attributes, is paramount for informing the development of TIS-targeted therapeutic strategies aimed at mitigating their deleterious effects on cancer treatment outcomes.

Secondly, the SASP produced by TISnt cells is also noted for its diverse and dynamic nature. SASP factors play a significant role in TME, contributing to both tumor progression and immune modulation. Key SASP factors, such as pro-inflammatory cytokines (e.g., IL-6, IL-8), growth factors (e.g., VEGF), and matrix remodeling enzymes (e.g., MMPs), have been shown to promote tumor progression and immune evasion by recruiting and activating immunosuppressive cells like M2 macrophages and regulatory T cells (Tregs). To effectively target TISnt cells and mitigate their detrimental effects, it is crucial to identify the key SASP factors involved in these processes (Fig. 5b). For instance, genomic profiling can be used to identify differentially expressed genes in TISnt cells, providing insights into the molecular pathways driving SASP production. RNA sequencing (RNA-seq) allows for comprehensive mRNA expression analysis, enabling the identification of novel SASP factors and their regulatory mechanisms. Proteomics, including mass spectrometry, can be employed to analyze protein expression and interactions, offering a deeper understanding of the signaling pathways activated in TISnt cells. Metabolomics can be used to profile metabolic alterations in TISnt cells, providing insights into how cellular metabolism supports SASP production and its role in tumor progression. Particularly, a key research priority is understanding how TISnt cells interact with and remodel the immune microenvironment, which is often immunosuppressive due to SASP factors. Investigating ways to fine-tune SASP to enhance immune clearance while mitigating its tumor-promoting effects remains a crucial research priority. By discerning the most critical components of the SASP, researchers can develop tailored therapeutic strategies designed to disrupt the protumorigenic effects exerted by TISnt cells. The use of senomorphics to selectively suppress deleterious SASP components while preserving immune-stimulatory factors could help achieve this balance.

Finally, while targeting TISnt cells holds promise for improving cancer therapy efficacy, potential side effects and unintended consequences must be carefully considered. Therapeutic interventions aimed at TISnt cells may inadvertently disrupt normal tissue function or trigger immune-related adverse events.^235^ Therefore, a balanced approach that weighs the benefits against potential risks is essential in the development and implementation of TISnt cell-targeted therapies (Fig. 5c). Senolytics are agents that selectively induce cell death of senescent cells, while senomorphics modulate the senescent cell phenotype to attenuate its deleterious effects. The employment of these compounds can be strategically optimized by leveraging insights into the predominant senescent cell populations and the functional composition of the SASP. For example, pan-mTOR inhibitors could synergistically enhance the senolytic activity of Navitoclax, effectively reducing the accumulation of TISnt cells and mitigating SASP in both cellular models and Drosophila.^236^ Furthermore, integrating strategies to enhance host immune responses offers a promising avenue to augment the effectiveness of senolytic and senomorphic therapies. Approaches such as immune checkpoint inhibitors, cytokine therapies, or vaccines targeting senescent cell-specific antigens can bolster the immune system’s capacity to identify and eliminate senescent cells. Identifying immunosuppressive biomarkers to distinguish TISnt cells from normal senescent cells could improve precision targeting. Additionally, agents targeting metabolic or epigenetic modifications may help reprogram senescent cells for immune clearance or apoptosis.^116,237^ These combinatorial strategies hold significant potential for the efficient clearance of TISnt cells, thereby overcoming therapeutic resistance.

Obviously, TISnt cells pose a considerable barrier to the long-term success of cancer treatment, particularly in head and neck cancers. However, they also represent a critical therapeutic target, offering opportunities to innovate treatment paradigms to overcome therapy resistance. How to develop “one-two punch” strategy more precisely holds significant promise to overcoming therapy resistance linked to TISnt cells. Computational modeling approaches are revolutionizing our understanding of TISnt cell dynamics and resistance mechanisms by bridging multiscale biological data with predictive analytics. For instance, Whole-cell models, such as those developed by Karr et al.^238^, can simulate genotype-to-phenotype relationships in TISnt cells, predicting how driver mutations (e.g., *TP53/CDKN2A*) alter metabolic and secretory profiles (e.g., NAD⁺ dependency, SASP heterogeneity) to sustain survival under therapy. 3D genome modeling platforms, such as OpenNucleome^239^ reveal that rare chromatin interactions; such as *NF-κB*-enhancer loops, may perpetuate pro-survival SASP signals despite senolytic pressure. Agent-based models (ABMs)^240^ can map spatial crosstalk between TIS cells and immune infiltrates, identifying optimal windows for combining senolytics (e.g., ABT-263) with checkpoint inhibitors. Furthermore, machine learning approaches can leverage published screening data to discover novel senolytic compounds. For example, oleandrin has shown greater potency and activity in targeting Na + /K+ATPase and its senolytic effector NOXA compared to known cardiac glycosides with senolytic properties.^241^ A machine learning-based tool, Senescent Cell Identification (SenCID), can accurately detect senescent cells in bulk and single-cell transcriptomic data, to distinguish senescence baselines, assess stemness, predict gene functions, and evaluate responses to senolytic treatments.^242^ These computational and machine learning strategies hold significant potential for advancing personalized cancer therapies by optimizing senescence-targeted treatments in HNC. In summary, by integrating these approaches, we can not only improve direct cancer cell eradication but also mitigate TIS-driven immunosuppression and recurrence. Continued research into intricate molecular mechanisms driving TISnt cell dynamics and therapy resistance is essential to optimize this strategy. Ultimately, such targeted, computationally guided approaches have the potential to transform therapeutic efficacy and clinical outcomes in HNC.
